# Targeting Glioblastoma Stem Cells: A Review on Biomarkers, Signal Pathways and Targeted Therapy

**DOI:** 10.3389/fonc.2021.701291

**Published:** 2021-07-08

**Authors:** Xuejia Tang, Chenghai Zuo, Pengchao Fang, Guojing Liu, Yongyi Qiu, Yi Huang, Rongrui Tang

**Affiliations:** ^1^ Department of Neurosurgery, University-Town Hospital of Chongqing Medical University, Chongqing, China; ^2^ Department of Pharmacy, College of Veterinary Medicine, Sichuan Agricultural University, Chengdu, China; ^3^ Department of Neurosurgery and Key Laboratory of Neurotrauma, Southwest Hospital, Third Military Medical University (Army Medical University), Chongqing, China; ^4^ Department of Neurosurgery, The Ninth People’s Hospital of Chongqing, Chongqing, China

**Keywords:** glioblastoma, glioblastoma stem cells, biomarkers, signal pathways, targeted therapy

## Abstract

Glioblastoma (GBM) remains the most lethal and common primary brain tumor, even after treatment with multiple therapies, such as surgical resection, chemotherapy, and radiation. Although great advances in medical development and improvements in therapeutic methods of GBM have led to a certain extension of the median survival time of patients, prognosis remains poor. The primary cause of its dismal outcomes is the high rate of tumor recurrence, which is closely related to its resistance to standard therapies. During the last decade, glioblastoma stem cells (GSCs) have been successfully isolated from GBM, and it has been demonstrated that these cells are likely to play an indispensable role in the formation, maintenance, and recurrence of GBM tumors, indicating that GSCs are a crucial target for treatment. Herein, we summarize the current knowledge regarding GSCs, their related signaling pathways, resistance mechanisms, crosstalk linking mechanisms, and microenvironment or niche. Subsequently, we present a framework of targeted therapy for GSCs based on direct strategies, including blockade of the pathways necessary to overcome resistance or prevent their function, promotion of GSC differentiation, virotherapy, and indirect strategies, including targeting the perivascular, hypoxic, and immune niches of the GSCs. In summary, targeting GSCs provides a tremendous opportunity for revolutionary approaches to improve the prognosis and therapy of GBM, despite a variety of challenges.

## Introduction

Malignant gliomas are the most frequent and lethal cancers originating in the central nervous system (1), and include three main high-grade glioma phenotypes based on genome-wide expression profiling and DNA methylation analysis in recent clinical assessments: proneural, mesenchymal, and classical (2, 3). Glioblastoma (GBM), also known as glioblastoma multiforme, is one of the most biologically aggressive subtypes due to its high malignancy and genetic heterogeneity (4, 5). The standard therapeutic approaches for GBM include maximal surgical resection, radiotherapy, chemotherapy (with various chemotherapeutic agents), and other innovative therapies combined with conventional approaches (6–8). Despite this, only 5.5% of patients between the ages of 55 and 64 survive for five years from diagnosis, and life expectancy has only increased from approximately 6 to 12–18 months after maximal standard treatments. This dismal prognosis is associated with the high rate of relapse and resistance to current standard therapies (9, 10). Overall, treatment of GBM is still challenging compared to other solid tumors, despite the great advances in medical development and improvements in therapeutic methods. Therefore, there is an urgent need to identify novel therapeutic targets to optimize the therapeutic approaches to GBM treatment.

Disease management and standard therapies are the absence of work due to the considerable intratumoral phenotype, intrinsic heterogeneity, and extensive infiltration of tumor cells throughout the brain (11). Thus far, several studies have identified the presence of stem cell-like cells in solid tumors, termed cancer stem cells (CSCs). These cells have high plasticity and the ability to proliferation, self-renew, and give rise to pathognomonic heterogeneous cancer cells that comprise the tumor (1, 12–14). A study on a pure CSC tumor model found that CSCs form the basis of tumorigenesis and continue to proliferate through self-renewal and segregation into different tumor cells (15). Simultaneously, numerous studies have shown that the highly malignant CSC subpopulation of tumor cells shows more aggressive potential compared to non-CSCs, despite the fact that the differences between CSCs and non-CSCs are not fully clear (16–18). A huge amount of evidence has shown that malignant CSC subpopulation of tumor cells in GBM are probably linked to malignant relapse, resistance to standard therapies due to their characteristic abilities to self-renewal, differentiation, growing and progression (12, 19–25). Therefore, CSCs causes of tumor characteristics such as proliferation, maintenance, malignant relapse, and metastasis in GBM (26). The major reasons for this resistance to chemo- and radiotherapy are their assumed quiescence, high capacity for extensive DNA repair, a higher mitochondrial reserve and their location in hypoxic niches (2, 27, 28). In order to combat therapeutic resistance of CSCs, it is necessary to understand both the mechanisms of inherent resistance and the surrounding niche of CSCs, which might be perceived as an interesting alternative to targeting therapy for GBM. It is also crucial to further understand the characteristics of these cells. First, CSCs must be identified prospectively from a variety of tumor cells. Common biomarkers, including CD133, CD44, and CD24, have been used to identify and enrich CSCs; however, several studies on CSCs have failed to confirm their reproducibility and accuracy owing to the genetic heterogeneity of CSCs, and the lack of universally informative biomarkers (29, 30). Furthermore, with the aim of treating this highly malignant disease, identification and blockade of CSC signaling pathways, such as the Notch, sonic hedgehog (SHH) and Wnt signaling pathways (31), which are closely correlated with tumor characteristics including proliferation, maintenance, malignant relapse, and metastasis in GBM, will provide vital targets for GBM treatment.

**Figure 1:**
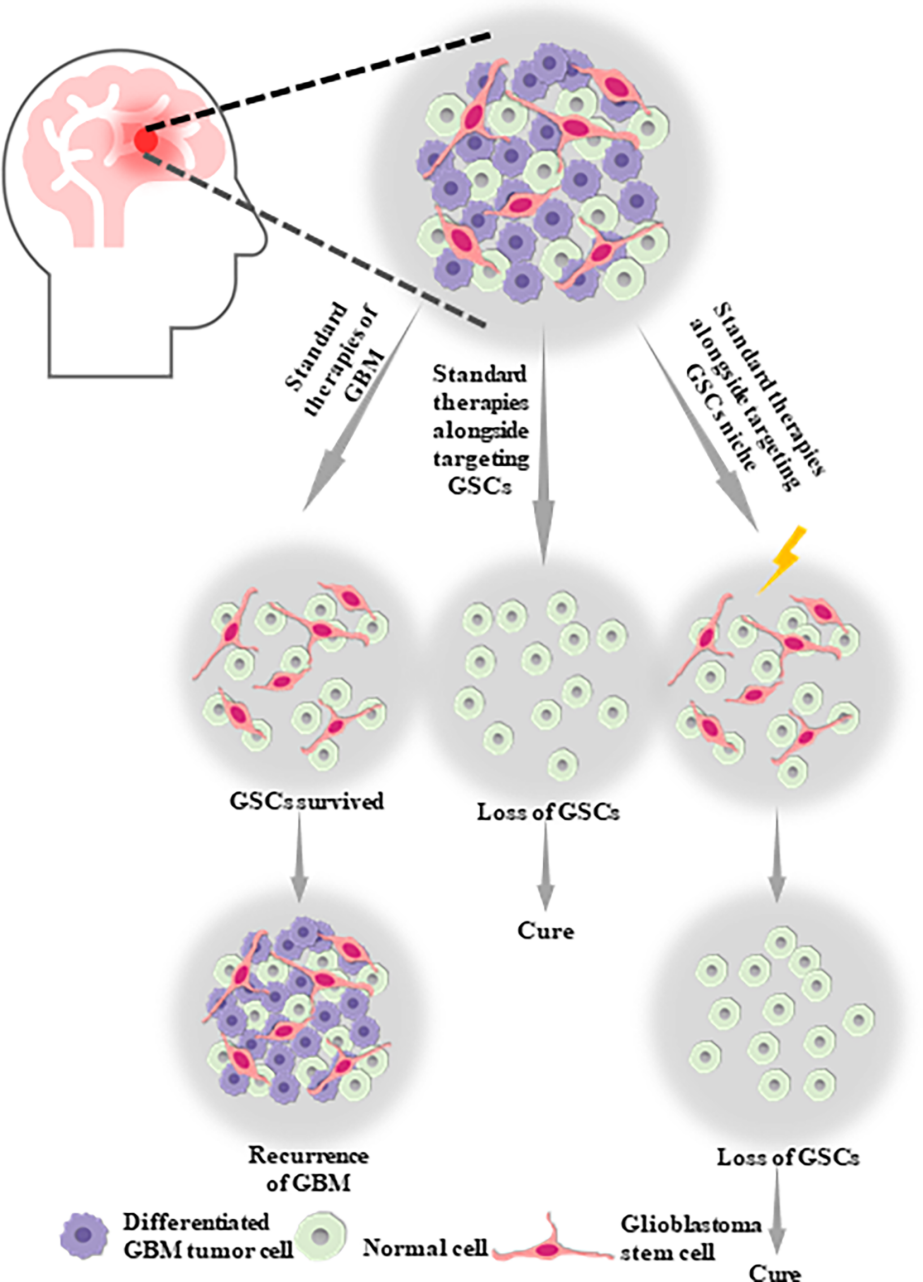
Glioblastoma stem cells (GSCs) are likely to be one of the potential causes of tumor proliferation, maintenance, malignant recurrence and metastasis in GBM, which aid in the self-renew, multilineage differentiation and proliferation of GSCs. Therefore, standard therapies alongside targeting GSCs or targeting GSCs niche might be the optimal therapy strategy for GBM.

In contrast to the bulk tumor populations, the unique properties of CSCs suggest that targeting the stemness of CSCs in GBM and developing targeted CSC therapies could offer an unprecedented therapeutic opportunity for GBM (**Figure 1**). This is important, as the refinement of the current standard therapy techniques for GBM is essential to improve the poor disease outcome. However, targeting CSCs for GBM therapeutics remains difficult, owing to the slow cycling of CSCs, the high expression of drug export proteins, and having no potential ability in CSCs to expressing the oncoproteins that could be targeted by the new generation of smart drugs, such as Gleevec and Iressa (32). In order to gain a better understanding of the characteristics of CSCs and CSCs-targeted therapeutic approaches to overcome therapeutic challenges, including the resistance and tumor recurrence in GBM, we summarize the current knowledge regarding glioblastoma stem cells (GSCs), their related signaling pathways, resistance mechanisms, crosstalk linking mechanisms, and microenvironment or niche. Subsequently, we present a framework of targeted therapy for GSCs based on direct strategies, including blockade of the pathways necessary to overcome resistance or prevent their function, promotion of GSC differentiation, virotherapy, and indirect strategies, including targeting the perivascular, hypoxic, and immune niches of the GSCs. In summary, targeting GSCs provides a tremendous opportunity for revolutionary approaches to improve the prognosis and therapy of GBM, despite a variety of challenges. We believe that this review could help to guide the future of GBM research and therapy.

## Neural Stem Cells and Glioma Stem Cells

### Neural Stem Cells

In the 1960s, James Till and Ernest McCulloch first discovered stem cells after performing modified spleen colony formation assays *in vivo* (33). The crucial capacity for undergoing asymmetric division *via* self-renewal of stem cells have been described, which could generate two daughter cells with different cell fates that is a true stem cell and the other one is a progenitor with a high cycle ability despite a limited number of cell cycles, and simultaneously the loss of this balanced process could induce a cancer-like state (34). Recently, stem cells as undifferentiated cells have been clearly defined that have the ability to undergo asymmetric division, malignant proliferation, self-renewal, plasticity, differentiation into progenitor cells or regeneration of injured tissue (35). The cell cultures of GBM have been verified to show capable of forming neurospheres *in vitro* with high expression of biomarkers like CD133 and Nestin (36), and these sphere-forming cells derived from human GBM into mice could also grow tumor *in vivo* (20). The same cell culture conditions could been used as for these normal neural stem cells (NSCs) (37), which have the ability to produce neural and glial cells of the nervous system, are one of the primary stem cell types in the nervous system, and the transformation from NSCs to GSCs has been demonstrated to be a possible factor in the formation of glioma (38). There are at least two neurogenic regions of the adult mammalian brain where NSCs are identified: the subventricular zone (SVZ) of the lateral ventricles and the subgranular zone (SGZ) of the hippocampus in the dentate gyrus. Stem cells near the SVZ or SGZ are always in a state of quiescence or active mitosis, as shown in several murine models (39–41). In addition, NSCs situated in the SVZ could also undergo asymmetric division as they are able to orient their division and their mitotic spindle acting as a molecular trigger for both daughter cells in the microenvironment (42–44). The malignant proliferation of NSCs has been proven to be one of the likely origins and identifying characteristics of cells that induce tumorigenesis, including proliferation, maintenance, malignant relapse, and metastasis in GBM due to the genetic mutations in oncogenes accumulation in NSCs, which may give rise to the dedifferentiation in normal brain cells and induce tumor cells (45–47).

### The Relationship Between NSCs and GSCs

These tumor cells possessing stem cell-like features seem to be responsible for tumor progression and tumor relapse in various solid tumors including GBM. The authentic origin of CSCs is a problem that has been extensively investigated, but some controversy about this question remains, due to the incomplete and inconsistent experimental data (48–51).

Interestingly, the tumor-propagating cells in GBM named GSCs have properties similar to those of normal NSCs, including the enhanced ability to self-renew and undergo multilineage segregation into different tumor cells (52, 53). In addition to these similar stem cell properties, the common pathways between GSCs and NSCs, such as Notch, bone morphogenic proteins(BMPs), Wnt, NF-κB, platelet-derived growth factor (PDGF), epidermal growth factor (EGF), SHH, and transforming growth factor–b (TGF-b) are closely linked to the development of the nervous system (36, 54, 55). Among these, PDGF signaling is also important for the transformation of normal NSCs into GSCs, facilitating the formation and proliferation of GBM (56–58). EGFR signaling has been demonstrated to play a pivotal role in regulating normal NSC expansion, migration, differentiation, and survival in neural development (59–62), inducing tumorigenic potential (63, 64). The similar gene expression profiles between NSCs and GSCs also support the hypothesis that CSCs are the other malignant variants of NSCs (65, 66). Furthermore, GSCs and NSCs express semblable neuronal or glial markers, such as CD133, CD15, integrin-α6, L1CAM and (67). In contrast to the remaining isolated cells which lack these markers, CSCs with common NSC markers have undergone orthotopic tumor formation in nude mice, and the orthotopic tumors are closely related to human GBM (68). In summary, there is plenty of evidence to indicate the common properties, signaling pathways, gene expression, and biomarkers between NSCs and GSCs, however, it is still difficult to confirm whether GSCs are derived from malignant mutation of NSCs, which could induce tumor formation, or whether they are derived from other mature cells with the ability to self-renew and differentiate into various tumor cells. A reliable approach to targeting GSCs for GBM treatment would only selectively eliminate GSCs after identifying and isolating GSCs from other NSCs. In regards to this problem, further studies on the relationship between NSCs and GSCs are needed.

### Isolation and Identification of GSCs

Specific and precise criteria play an indispensable role in identifying and isolating GSCs from other cells (69). GSCs have been isolated from solid tumors and classified as one of the first cell types (23). Conversely, GSCs are effective at initiating tumor xenografts *in vivo*, owing to the formation of heterogeneous tumors that resemble the original parent tumor. On the contrary, GSCs have been demonstrated to self-renew and form neurospheres when grown *in vitro*, which could be used to assess the proliferation of GSCs *via* the frequencies of GSCs in tumors (70, 71). Consequently, GSCs cultured *in vivo* or *in vitro* play an important role in the pathogenesis and development of GBM, because of the greater reliability and physiological relevance of the model.

The use of various specific surface markers or molecular mediators such as CD133, CD90, CD44, L1CAM, A2B5, and GPD1 is a common method to identify GSCs and define lineage-specific subpopulations within the tumor, although the cause of their reproducibility and accuracy is still unclear (29, 30, 72). However, the GSC niche as a potential marker source may cause epigenetic changes and variations in CSC phenotypes *via* microenvironmental signals *in vivo* or *in vitro* (31). The recognition of GSCs as therapeutically targetable remains a major challenge to overcome. Furthermore, some stem cells might additionally express ABC transporters binding with ATP, which are able to pump the fluorescent dye HOECHST-33342 out of the cell and to recognize unlabeled side population (SP) highly enriched in stem cells in neural tissues (67), which could be an alternative approach for identifying GSCs. Another approach to identify a subpopulation of GSCs is the observation of autofluorescence around 520 nm under laser excitation at 488 nm, owing to the autofluorescence properties and distinctive morphology of GSCs (73). In summary, various strategies for the isolation and identification of GSCs have been produced, and have facilitated efforts to isolate and identify GSCs *via* new and more effective technology, which may require a thorough insight into both the essential molecular and morphogenic processes regarding how GSCs are associated with tumor initiation or tumor proliferation in GBM. To improve the diagnostic and therapeutic prediction value, it is important to develop methods that are more effective to isolate and identify GSCs in GBM.

### Biomarkers of GSCs

The isolation and identification of GSCs in GBM may contribute to a better understanding of the mechanisms underlying tumorigenesis and novel therapeutic strategies for GBM. It may be an ideal and effective approach to sort and target GSC populations *via* GSC markers to distinguish the expression of stem cell surface markers in GSCs, and are functionally correlated with the maintenance of GSCs, providing a powerful tool to investigate the tumorigenic process in the cerebral nervous system and promote the diagnosis, and treatment of GBM. To date, a great number of advances have been made in understanding GSC markers in the last several years. Here, we focus on several putative GSC markers, including CD133 (PROM-1), Nestin, LGR5, B23 (NPM1), and GPD1.

#### CD133 (PROM-1)

CD133, as the initial isolation marker of GSCs, is still the best validated marker. CD133, which belongs to the Pentaspan transmembrane glycoprotein family member, is also known as prominin-1 (PROM-1) or AC133 (74). It is a membrane bound glycoprotein encoded by the PROM1 gene and the chemical structure of CD133 involves a single-chain polypeptide of 865 amino acids with 5-TM regions, an extracellular N-terminus and a cytoplasmic C-terminus (75). CD133 was first discovered in human hematopoietic progenitor cells and was later described in mouse tissue (76). With the aim of enriching and identifying tumor-propagating and tumor-initiating cells, CD133 has become an alternative tool in some experiments. For example, CD133^+^ cells in GBM, identified by flow cytometry, have been considered tumor-initiating cells and have been demonstrated to be responsible for the proliferation of tumor cells, as well as resistance to radiation therapy based on immune-deficient NOD/SCID mouse xenograft models (77). However, there are still some controversies regarding CD133^+^ cells. Some studies have questioned the idea that CD133 as a stem cell surface marker is a common and useful tool for identifying and defining GSCs, including the findings that CD133^+^ cells are differentiated, while CD133^−^ cells also have the ability to initiate tumors in some cases (78), indicating that CD133 might not be a reliable GSC informative marker. There are still some debates regarding the definitive function of CD133 in GBM, but it is clear that the expression of CD133 may change due to several interactions with the tumor microenvironment, and may play a possible role in cell differentiation and the epithelial to mesenchymal transition (79). Given the limited knowledge regarding the definitive role of CD133 and its expression across the differentiation spectrum of GSCs, it is necessary to have a thorough insight into the function of CD133 in GBM.

#### Nestin

The neuroepithelial stem cell protein commonly known as nestin, first described as an antigen of rat-401 against embryonic spinal cord and later identified as a class VI intermediate filament protein (80), has been shown to be expressed in neuroepithelial stem cells, and is highly expressed in several types of human malignancies, including higher grade GBM. Nestin has been shown to be strongly correlated with lower cancer patient survival, while some researchers hold the opposing view that there is no connection between Nestin expression and poor prognosis in GBM (78, 81–85). Nestin, as another putative marker for the GSC phenotype, probably plays a significant role in aggressive growth metastasis and self-renewal capacity of CSCs, organizing the cytoskeleton, cell signaling, organogenesis, and cell metabolism, and represents the proliferation, migration, and multi-differentiated characteristics of multi-lineage progenitor cells, and it is thus a more suitable target molecule to identify GSCs in GBM than CD133 (86, 87). Zhang et al. previously showed that there have been considerable refinements in clinical prognostic accuracy by adding a putative marker for the GSCs Nestin into CD133 (88). Although a definitive description of Nestin in GSCs remains elusive, its role in transformed cells, especially GSCs, its involvement in forming the cytoskeleton and the thorough mechanisms underlying this relationship between Nestin expression and miscellaneous capacity of GSCs is known. Thus, a combined detection of Nestin and CD133 co-expression may be a potential indicator of the biological invasion of GBM.

#### LGR5

Leucine-rich repeat-containing G protein-coupled receptor 5 (LGR5) was first reported as a marker of intestinal stem cells and also serves as a novel functional marker of GSCs. LGR5 belongs to the seven-transmembrane receptor subclass of the G protein-coupled receptor family, is closely correlated with the Wnt signaling pathway and is also known as Gpr49 (89). LGR5 contributes to tumor formation, proliferation, and aggressiveness (90–94). In 2018, Zhang et al.demonstrated that LGR5 has a variety of roles, including promoting GSC epithelial-mesenchymal transition (EMT) by activating the Wnt/β-catenin pathway *in vitro* and *in vivo*, and should be an effective indicator of prognosis in GBM, along with predicting glioma recurrence (95). Therefore, targeting LGR5 in GSCs may facilitate the treatment of GBM with ideal therapeutic approaches to promote the further understanding of the novel functional marker of GSCs.

#### B23

B23, also known as nucleophosmin or NPM1, is both a chaperone of nucleic acids and a nucleolar protein shuttling between the nucleoli, nucleoplasm, and cytoplasm, and is involved in various functions, including centrosome duplication, ribosome maturation and export, intracellular transport, chromatin remodeling (core and linker histone binding), apoptosis, and mRNA splicing in diverse cellular processes, and plays an important role in the cellular response to different stress stimuli and cell cycle control (96, 97). It has been found that B23 is overexpressed at both the mRNA and protein levels in hematologic malignancies and other types of cancer compared with the normal brain, especially in gliomas (98). Consequently, B23 is considered a promising therapeutic target for GBM treatment.

#### Glycerol-3-Phosphate Dehydrogenase 1 (GPD1)

GPD1, also named as GPD-C, GPDH-C, or HTGTI, is one of three isoenzymes of human glycerol-3-phosphate dehydrogenase that are involved in catalyzing the conversion of dihydroxyacetone phosphate (DHAP) with NADH and glycerol-3-phosphate (G3P) with NAD^+^, which is critically implicated in the transport of reducing equivalents across the mitochondrial membrane and triacylglycerol synthesis (99–101). Scientists have demonstrated that aberrant GPD1 expression is found in dormant GSCs, but not in NSCs *in vivo*, and its expression could aggravate the progression of GBM due to its significance for GSC proliferation and maintenance, furnishing dormant GSCs with functional correlation as a therapeutic target (102). Therefore, loss of GPD1 could result in impairment of GSC maintenance pathways in GBM, prolonging patient survival. Furthermore, it is better to shed light on the crosstalk between GPD1 and edema in GBM, as edema occurs in patients suffering from human GBM (103).

Although growing putative GSC markers like CD133, CD90, CD15, A2B5, ALDH1, Label-retention, Nestin, proteasome activity, and ABC transporters (78, 104) have been used to recognize GSCs, and a considerable number of studies have been conducted on these markers to provide compelling and informative evidence that these putative GSC markers are powerful tools to investigate the tumorigenic process in the cerebral nervous system and promote the diagnosis and treatment of GBM (**Table 1**). However, the exact mechanisms and functionalities of these putative markers in GSCs have not been thoroughly elucidated. Given this problem, it is crucial to have a thorough and wide insight into identifying both specific markers of GSCs and the molecular mechanisms for the development of improved and tailored targeting of GSC treatments for GBM, especially paying closer attention to the various ideas of GSC isolation and identification, such as the concomitant use of different stem cell markers, instead of a single marker.

**Table 1:** Common markers and novel biomarkers on GSCs.

	CD133	CD44	Musashi-1	CD15	L1CAM	Integrin α6	Nestin	CD36	A2B5	LGR5	B23	GPD1
Category	Member of the pantaspanglycoprotein family	Glycoprotein	RNA-binding protein (RBP)	Fucose-containing trisaccharide	Glycoprotein	Member of the and cell-extracellular matrix adhesion molecules family	Intermediate filament	Scavenger receptor	Ganglioside	Member of the G protein-coupled receptor family	Phosphoprotein nucleoli	Isoenzymes of human glycerol-3-phosphate dehydrogenase
Origin	Hematopoietic stem cells, endothelial progenitors, myogenic cells and stem cells	Stem cells	Neuronal stem cells	Neural stem/progenitor cells and embryonic stem cells	Neural cells	Embryonic, hematopoeitic, and neural stem cells	Mammalian CNS stem cells during development	Microglia, endothelial cells, astrocytes, and neurons	Early stage of gliomagenesis and tumor propagation	Stem cells	The cellular response to different stress stimuli and cell cycle control, tumorigenes related to chromosome translocations or mutations	Dormant brain tumor stem cells (BTSCs)
Function	Effects on cell polarity, migration, stem cell-adjacent cell interactions, and ECM	Moderates homing of stem cells as an adhesion molecule	Inhibition of mRNAs translation and activation of target mRNA translation	Highly tumorigenic, differentiates into cells expressing glial and neuronal markers, and generates the cell heterogeneity of the primary tumor	Tumor growth, GSC radiosensitivity and DNA damage response regulation	Ensures proper neural stem cell division, enrichment for the GSC population, contributes to tumor cell proliferation, survival, self-renewal, and in *vivo* growth	Prompts tumor cell growth metastasis and GSCs self-renewal	Immune activation, lipogenesis for GBM cell growth	Specific progenitor cell marker	Tumor formation, proliferation and aggressiveness, predicts glioma recurrence	The transport of reducing equivalents across mitochondrion membrane and triacylglycerol synthesis, promotes the progression of GBM *via* GSC proliferation and maintenance	The transport of reducing equivalents across mitochondrion membrane and triacylglycerol synthesis, promotes the progression of GBM *via* GSC maintenance
Reference	(75)	(105)	(106, 107)	(24)	(108, 109)	(21)	(87)	(110)	(111)	(92)	(96)	(102)

## The GBM Niche

Niches, clearly defined as the tumor microenvironment (TME) composed of extracellular matrix (ECM) and a complex tissue of cells including astrocytes, macrophages, pericytes, fibroblasts, and endothelial cells play a significant role in stem cells that not only an anatomic structural unit surrounding stem cells, but also a functional unit that provides complex and dynamic interactions with stem cells (13, 112). In the human brain, stem cells, including normal NSCs, are enriched in specific regions that consist of endothelial and ependymal cells to maintain their stem cell properties (14). The relationship between TME and stem cells is synergistic and codependent like the ‘‘seed-and-soil’’ relationship rather than passive relationship, which are adapted in GSCs, as they also require a specific niche to support their stem-like characteristics, including division other than self-renewal (113), high proliferation (114), and multidirectional differentiation into various tumor cells. In addition, multilineage and inherent crosstalk between GSCs and microenvironment with various tumor components such as the (ECM), cellular compartments such as cancer-associated fibroblasts, immune cells, differentiated neural cells, etc., and the blood–brain barrier (BBB) through tumor-derived pericytes is facilitated to form an ideal microenvironment that could enhance the invasive tumor-properties, or resistance to chemotherapy and radiotherapy (79, 115). The chemo- and radio- resistance of GBM is thus responsible for an isolated properties of GSCs, along with the intrinsic dependance on the synergistic interaction between these tumor cells and TME. These properties of the GSCs niche could be conducive to GBM heterogeneity, plasticity, and malignancy, determining the fate of GSCs.

There are three major GSC niches, including the perivascular niche *via* angiogenic pathways, the perinecrotic or hypoxic niche *via* inducing hypoxia, and the immune niche. The perivascular niche is surrounded by a mass of blood vessels feeding the tumor with abnormal structure and function. The synergistic and codependent relationship between the GBM perivascular niche and GSCs can contribute to the progression of tumor angiogenesis (116). There are a great number of CSCs under hypoxic conditions, which indicates a positive player in maintenance of CSCs through supporting the critical stem cell traits of multipotency, self-renewal, and tumorigenicity (117). HIFs(HIF-1α and HIF-2α) in the hypoxic niche are upregulated *via* the expression of HIF-1α and HIF-2α isoforms in GBM and the roles of both HIF-1α and HIF-2α seem to be overlapping with 75% homologies between HIF-1α and HIF-2α (118). In addition, it has been found that tumor aggression increased *via* acting on the GSCs and the infiltration of a large number of immune cells in the GBM immune niche, showing that the hypoxic response along with inflammation are overlapping despite the immune privilege in the normal human brain (119). The hypoxic environments could induce synthesis of HIF-1α with the protein stabilized through engaging the T-cell receptor (120), enhance the lytic ability of CD8+ T lymphocytes (121) as well as interferon-gamma secretion by CD4+ T cells (122), impair cytotoxic T lymphocyte (CTL) development, proliferation and expression of inflammatory cytokines (121), and also recruit immunosuppressive cells *via* GSCs signaling including tumor-associated macrophages (TAMs), myeloid-derived suppressor cells (MDSCs), and Tregs that could promote angiogenesis, inhibit the immune response by secreting chemokines and growth factors such as vascular endothelial growth factor (VEGF), transforming growth factor-β1 (TGF-β1), neurotensin, SDF1, and soluble colony-stimulating factor 1 (sCSF-1) and expressing surface molecules that engage inhibitory molecules on effector immune cells (123), ultimately leading to the formation of an immunosuppressive microenvironment. In a word, targeting of the GSC niche, particularly the perivascular niche, the hypoxic niche, and the immune niche, is still one of the crucial strategies to solve resistance to current standard therapy for GBM and improve the poor disease prognosis of GBM.

## Direct Targeting of GSCs

### Blockage of GSC Signaling Pathways

A sequence of signaling pathways and receptors upregulated by GSCs involved in tumor proliferation, maintenance, and resistance to chemotherapy and radiotherapy, could improve the stem-like features and aberrant cell survival, ultimately giving rise to oncogenesis in the brain (36). It is thus crucial to acquire a thorough insight into the pivotal signaling pathways and receptors responsible for GSC maintenance, including Notch, Wnt, SHH and Receptor Tyrosine Kinase (RTK) pathways, to understand the stem cell properties of GSCs and facilitate the development of improved and tailored targeting GSCs treatments for GBM.

#### Blockage of Notch Signaling Pathway

It has been previously confirmed that the Notch signaling pathway is critically associated with stem cell fate determination, proliferation, maintenance of cell quiescence, metastasis, and modulation of differentiation of both normal NSCs and GSCs (124). Via mutual effects with ligands on a considerable number of cells, the Notch pathway, including the four Notch receptors (Notch 1–Notch 4) and five ligands (Jagged-1 and Jagged-2, and Delta-like-1, Delta-like-3, and Delta-like-4), can regulate the interaction between cells and neighboring cells covering a short range (125, 126). The Notch signaling pathway is activated by sequential proteolytic cleavage reactions, causing release and nuclear translocation of the intracellular domains of Notch receptors (NICDs) and a series of transcription-dependent pathways, activated by the Notch signaling pathway are essential (127). Inhibitors of the γ-secretase (GSIs) complex which participates in regulating the last proteolytic step for release of NICDs, such as RO4929097 (29), play a significant role in blocking the Notch signaling pathway both *in vitro* and *in vivo*, owing to its ability to activating the Notch signaling pathway (128).

#### Blockage of Wnt/β-Catenin Signaling Pathway

The Wnt/β-catenin signaling pathway is critically implicated in modulating the differentiation and proliferation of normal neural cells such as NSCs or cells of the astroglial lineage (129–131), as well as the self-renewal, differentiation, and expansion of GSCs (132–134). The nuclear localization of stabilized β-catenin is able to abnormally activate the β-catenin signaling pathway of GSCs, and is intrinsically correlated to tumorigenesis, growth invasion in GBM (135, 136), and the expression of MGMT, which is responsible for the resistance to TMZ (137). However, it remains highly difficult to target the Wnt/β-catenin signaling pathway owing to the serious side effects of this inhibition, as this pathway is critically related to a considerable number of physiological processes in human organs including the brain (138).

#### Blockage of SHH Signaling Pathway

It has been well demonstrated that normal NSC fate determination, proliferation, maintenance, differentiation, and ventral patterning in the special and pivotal region called SVZ is intrinsically dependent on the SHH pathway in the adult brain and organogenesis, such as neural progenitor modulation during embryonic development (36). The SHH signaling pathway is critically implicated in the oncogenesis of GBM and the self-renewal of GSCs, as the activation of the SHH signaling pathway upregulates the drug efflux Pglycoprotein (ABCB1), ABC transporter ABCG2, multi-drug resistance-associated protein-1(ABCC1/MRP1), MGMT, B cell-specific Moloney murine leukemia virus integration site 1 (BMI1) (31, 139–141). Furthermore, the loss of P53 causes up-regulation of the potent transcription factor Nanog, which is closely correlated with SHH signaling pathway activity and the ability to regulate GSC properties, eventually contributing to chemo-resistance to TMZ in GSCs *via* regulating the expression of the TMZ resistance marker MGMT (141) and maintaining the self-renewal, differentiation, and expansion of GSCs based on recent studies (71).

#### Inhibition of Receptor Tyrosine Kinase (RTK) Pathways

RTK pathways are transmembrane proteins composed of a unique extracellular ligand-binding domain, a transmembrane helix, an intracellular tyrosine kinase domain, and a series of tyrosine residues (142, 143). RTK pathways include some of the most extensively studied pathways in oncology; here, we will focus on several important growth factor receptors in GBM and the targeted strategies to inhibit them.

#### Epidermal Growth Factor Receptor (EGFR)

The epidermal growth factor receptor (EGFR) is a transmembrane RTK that is critically responsible for the modulation of stem cell expansion, metastasis, differentiation, and survival in the human brain (144). In the case of heterodimers or homodimers formed by binding to ligands consisting of EGF or transforming growth factor-α (TGF-α), EGFR causes autophosphorylation of its C-terminal tail and activation of downstream signaling *via* its docking site of the SRC homology domain (145). It has been found that the over-expression of EGFR in humans accounts for 40%–60% of primary GBM tumors, especially in the classical subtype (146), however, the over-expression of EGFR in GBM is intrinsically implicated in gain-of-function missense mutations and in-frame deletions in the extracellular domain, rather than responsiveness to EGFR inhibitors (147, 148). The transactivation of β-catenin enables EGFR activation, leading to GSC expansion, metastasis, differentiation, tumor formation (149), and subsequently the human oncogenic EGFR is over-expressed, enhancing the self-renewal ability of GSCs, and further contributing to tumor- initiation or tumor- proliferation in GBM (36, 63, 64). Furthermore, in a GSCs marker study conducted by Song et al. (150), in 2021, SH3KBP1, a promising therapeutic target in GBM patients, was demonstrated to have the capacity to activate and modulate EGFR signaling. There are three kinds of EGFR tyrosine kinase inhibitors (TKIs) in total, including first-generation reversible small-molecule TKIs, which target EGFR and its co-receptor HER2, such as Erlotinib and Gefitinib (151), second-generation TKIs which bind irreversibly to EGFR, such as Afatinib, Neratinib and Dacomitinib (138) and third-generation irreversible inhibitors such as TKIs AZD9291 (Osimertinib), which have excellent blood–brain barrier penetration and have been shown to be effective in preclinical tests (152).

#### Platelet Derived Growth Factor Receptor (PDGFR)

The PDGF family of receptors, one of the main deregulated RTK pathways in GBM, is comprised of two categories of receptors by different genes encoding PDGFRα, which is mainly responsible for developing oligodendrocytes (153) and PDGFRβ, which is important for blood vessel formation (154) during embryonic development. It has been found that the activation of PDGFR-signaling *via* a PDGF-nitric oxide (NO)-ID4-regulatory circuit (155) in humans accounts for tumorigenesis in 30% of GBM patients, and the overexpression or alterations of PDGF ligands are commonly found in GBM patients, especially in a proneural subtype of GBM, which contributes to the development of GBM and the function of GSCs, such as the self-renewal and tumor-initiating capacity to different extents among PDGFRα and PDGFRβ (155). Among several PDGF inhibitors, such as imatinib, tandutinib, AG1433, and nilotinib (138, 156), the small molecule TKI imatinib (also known as Gleevec; Glivec; STI-571) has been demonstrated to enable the growth of GBM xenografts *in vivo* to inhibit and the radiosensitivity of human GSCs *in vitro* to increase (157), but it has no significant therapeutic effect on recurrent GBM (156).

#### Vascular Endothelial Growth Factor Receptor (VEGFR)

Three RTKs, VEGFR1, VEGFR2, and VEGFR3, which are encoded by the genes FLT1, KDR, and FLT4, constitute the VEGF receptor (VEGFR) family (158–160), a pivotal regulator of vasculogenesis, angiogenesis, and lymphangiogenesis (VEGFR3) in the normal body (161). GBM is one of various tumors which highly expresses VEGF and its receptors, resulting in highly vascularized tumors and increased microvasculature compared to other normal tissues (162), and is an important target in glioblastoma aberrant VEGFR2 signaling, which is an important pathway affecting survival, proliferation, migration, and vessel permeability in tumor cells (163). Furthermore, it has recently been demonstrated that human cartilage glycoprotein-39 or chitinase-like protein-1 (YKL-40) may be effective targets, as they are able to upregulate VEGF expression and induce new tumor vasculature (164). There are a wide variety of VEGFR inhibitors (TKIs) with different treatment effects, including atalanib (PTK787), mainly against VEGFR2, PDGFR, and c-kit (165), sorafenib (166), tivozanib (167), pazopanib combined with lapatinib (168), Cediranib (169), and SU1498 (170).

Therapeutic targeting of GSC pathways and receptors, such as Notch, Wnt, SHH, EGFR, PDGFR, and VEGFR presented in this review, which are critically implicated in tumor cell proliferation, maintenance, and resistance to current therapies, is of significant interest as it provides reliable and physiologically relevant trials to block these stem cells *via* the inhibition of pathways and receptors. Among these, EGFR, PDGFR, and VEGFR inhibitors have shown less advantages for their limitations compared to GSC pathways, such as Notch, Wnt, and SHH pathways, suggesting that targeting GSC pathways might become a future direction and an excellent solution in GBM therapy.

### Promotion of GSC Differentiation

Multilineage differentiation both *in vitro* and *in vivo* is an important property in NSCs, and NSCs share the ability to promote cell differentiation in common with GSCs (68). In addition, GSCs have been proven to become inherently more sensitive to therapy, less capable of engraftment, and enable apoptosis to be directly induced in some settings after differentiating into more terminal glioblastoma cells (2). Hence, the promotion of GSC differentiation might be a key player in the therapeutic strategies for GBM. The bone morphogenic proteins (BMPs) involved in promoting normal neural precursor differentiation into astrocytes and post-transcriptional modification using miRNA, have been demonstrated to play a positive role in the stem cell niche of the adult brain, increase in GSC differentiation, and suppression of glioblastoma tumorigenicity *in vivo*, leading to the elimination of GSCs and sensitization of GBM to chemotherapeutics (2, 171). Recently, the overexpression of miR-128 or miR-302a has been shown to promote GSC differentiation, enhance senescence mediated by axitinib treatment, and further impair GSC proliferation (172). Furthermore, graphene oxide (GO), a new carbon material, has been reported to have potential for use in GBM treatment, as GO could decrease the expression of stem cell markers such as SOX2 and CD133, and increase the expression of differentiation-related markers such as GFAP and β-III tubulin, ultimately inducing the differentiation of GSCs (173). Sulindac, a non-steroidal anti-inflammatory drug (NSAID), is capable of inducing GSC differentiation and sensitizing them to oxidative stress (174).In the future, novel approaches for the promotion of GSC differentiation and potent anti-GBM agents such as GO and Sulindac, which could be used to suppress GSCs and become useful for future clinical applications, are urgently needed.

### Virotherapy

As strictly intracellular organisms, viruses can replicate inside host cells by hijacking the cellular machinery. Among these various targeting GSC strategies, virotherapy has shown therapeutic value in terms of preventing GBM recurrence; here, we will shed light on oncolytic virotherapy (OV), which has been extensively studied. Oncolytic virotherapy possesses lytic properties and is capable of achieving tumor cell lysis through intra-neoplastic virus replication (175). Several approaches for delivering OV with stem cells against recurrent GBM have been studied, including intra-arterial delivery of allogeneic bone marrow-derived human mesenchymal stem cells loaded with the oncolytic adenovirus DNX-2401 (BM-hMSCs-DNX2401), and injection of NSCs to deliver an oncolytic adenovirus into newly diagnosed GBM (4). Furthermore, this might be an ideal strategy to overcome the limitations of oncolytic viral vectors, such as limited biodistribution, dismal replication, and negligible transduction of neighboring tumor cells after intracranial injection to study some vectors that possess the capacity to specifically target the small population of GSCs with resistance to chemotherapy and radiotherapy, in addition to more globally targeting the bulk tumor populations.

## Indirect Targeting of GSCs *via* the GBM Niche

**Figure 2:**
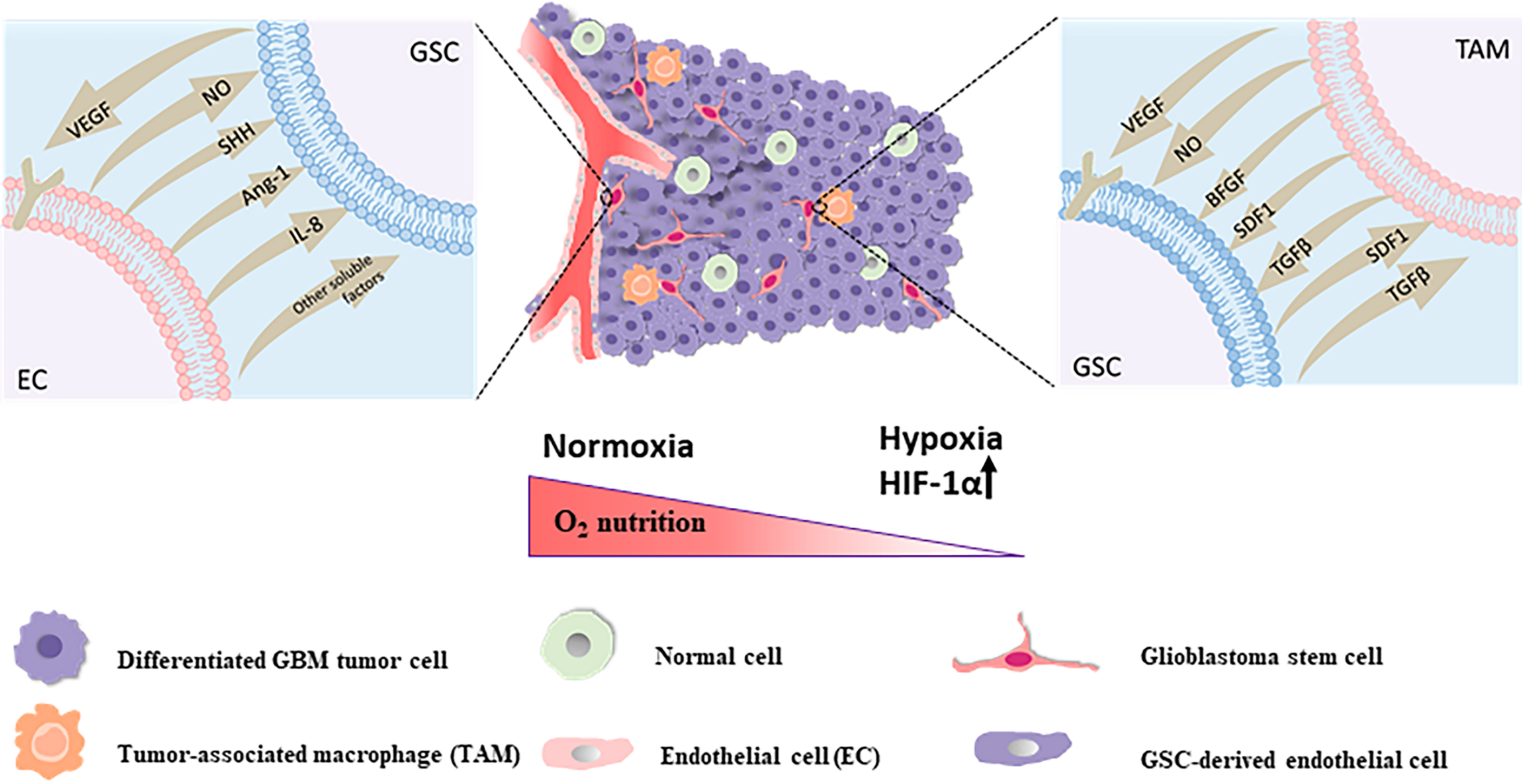
The perivascular niche The self-renewal and maintenance of neighboring GSCs within the perivascular maintenance niche could been promoted *via* secretion of endothelial-derived diffusible signals including nitric oxide (NO), SHH, angiopoietin-1 (Ang-1), IL-8 and other soluble factors. GSCs are capable of stimulating the proliferation of endothelial cells and the sprouting of new vessels *via* secretion of VEGF and SDF-1 in the local tumor environment for sustainability and expansion of the vascular maintenance niche. The hypoxic niche With the constant invasion of GSCs, activation of hypoxia-related factors might take an active role in stemcell maintenance and one of the main factors is the hypoxia response Hypoxia Inducible Factor 1α (HIF-1α). The immune niche On the one hand, for TAMs, GSCs could chemo-attract and recruit TAMs to the tumor site, prompt the growth of macrophages and induce the polarization of TAMs into the immunosuppressive M2 phenotype through secreting chemokines and growth factors like VEGF, transforming growth factor-β (TGF-β), SDF1 and soluble colony-stimulating factor 1 (sCSF-1). On the other hand, the accumulation of these pro-tumorigenic TAMs in tumor could secrete cytokines and signaling molecules such as basic fibroblast growth factor (bFGF), TGFβ, SDF1, VEGF, and nitric oxide (NO), contributing to tumor progression and GSCs maintenance in turn.

There are three major methods of indirect targeting of the GSC niche, including targeting of: the perivascular niche *via* angiogenic pathways, the perinecrotic or hypoxic niche *via* inducing hypoxia, and the immune niche (**Figure 2**), which is still one of the crucial strategies to solve resistance to current standard therapy for GBM and improve the poor disease prognosis of GBM.

### Targeting the Perivascular Niche

The perivascular niche is surrounded by a mass of blood vessels feeding the tumor with abnormal structure and function, including poor and irregular formation (leaky and friable blood vessels), hypoxia, loss of hierarchy, and an impaired blood–brain barrier (BBB). This vasculature is distinct from the normal blood vessels in humans, which are formed primarily through vasculogenesis and angiogenesis, and transport gases, nutrients, and waste products for the human body (176, 177). The synergistic and codependent relationship between the GBM perivascular niche and GSCs has been extensively proven. For example, it is known that GBM mediates the perivascular region to maintain GSCs survival, proliferation, and migration (178), simultaneously GSCs act as a regulator of the cancer-specific vasculature, infiltrating the tumor and thus contributing to the progression of tumor angiogenesis (116). High levels of angiogenic factors produced by GSCs in the human body are conducive to the abnormal formation of tumor blood vessels under hypoxic microenvironments, including vascular endothelial growth factor (VEGF), SDF-1, PDGF, and fibroblast growth factor (FGF), providing beneficial conditions for GSC survival and expansion (178–180). Consequently, this might become an ideal approach for GBM therapy by indirectly targeting the perivascular niche of GSCs and interfering in the aberrant vascular proliferation *via* the inhibitors angiogenic factors in tumor and simultaneously combined with other treatments that might inhibit progression of tumor malignancy or GSC differentiation, such as in combination with immunotherapeutics, especially those designed to repolarize macrophages (181) to overcome the challenge that malignant progression in GBM is prompted by increasing the invasion and metastasis of tumor cells after the blockage of angiogenesis that could shrink the original tumor (14).

### Targeting the Hypoxic Niche

Hypoxia as one of the diagnostic hallmarks of GBM, with median oxygen saturation levels of <2% in necrotic regions, compared to the normal median oxygen concentrations of approximately 5% in physiological tissues, owing to vigorous metabolic activity and plentiful oxygen consumption in heterogeneous tumor cells, along with chaotic and poor functioning blood vessels (182) that are able to maintain and promote the intrinsic cellular function of GSCs involved in self-renewal and differentiation, as well as enhance angiogenesis, tumor aggression, and chemo- and radiotherapy resistance in GBM, which are the main reasons for GBM patient death (183, 184). It has been demonstrated that one of the main regulators of the hypoxia response Hypoxia Inducible Factor-1α (HIF-1α) and Vascular Endothelial Growth Factor A (VEGFA) staining and tumor vascularity are critically implicated in worse progression-free survival and are responsible for lower patient survival rates of GBM based on dynamic contrast-enhanced MRI analyses (185, 186), while another hypoxia inducible factor (HIF) family HIF-2α indicates an important role of maintaining GSC survival and proliferation, as blockage of HIF-2α *via* the gene silencing technology or others would significantly compromise the intrinsic cellular features of GSCs (26, 187). Moreover, VEGFA is one of the main factors prompting the invasion of GSCs and polarizing immune cells into an immunosuppressive phenotype, causing treatment resistance to both standard and modern approaches *via* tumor-associated macrophage M2 polarization, increased regulatory T cells, and higher rates of PD-1^+^ CD8^+^ T cells (188–191). Bevacizumab, a humanized monoclonal antibody and inhibitor of VEGFA, is thus a promising anti-VEGF agent to create persistent normalization through pruning vessels and overcoming the recurrence of hypoxia and the emergence of resistance in GBM (192).

### Targeting the Immune Niche

In GBM mouse model systems, scientists found that tumor aggression increased and the infiltration of a large number of immune cells, such as TAMs and other CD11b^+^ myeloid cells emerged in the brain after treating recurrent GBM patients with bevacizumab or the angiokinase inhibitor cediranib, despite the immune privilege in the normal human brain (119, 193–195). The direct interaction of GSCs with these immune cells could contribute to resistance of antiangiogenic therapy by generating VEGF-independent angiogenesis and immunosuppression, which leads to the development of tumors lacking the ability to respond to VEGF inhibition (196, 197). TAMs are the most prevalent tumor-infiltrating inflammatory cells in GBM (188, 189). A considerable number of infiltrating TAMs in the GSC niche, including the blood-derived macrophages and resident microglia (198), indicates a pivotal player in GBM tumor progression and GSC maintenance by a pleiotrophin–PTPRZ1 signaling axis (199), and is also positively linked to the poor prognosis of GBM and the high malignancy grade of glioma (200, 201). The relationship between the GBM immune niche and GSCs is also synergistic and interactive. On the one hand, for TAMs, GSCs could facilitate chemo-attraction and recruit TAMs to the tumor site upon the hypoxic conditions, prompt the growth of macrophages, and induce the polarization of TAMs into the immunosuppressive M2 phenotype by secreting chemokines and growth factors such as VEGF, TGF-β1, neurotensin, SDF1, and sCSF-1 (202–206). Additionally, for T lymphocytes, GSCs play a direct role in inhibiting the proliferation and activation of T cells and inducing T cell apoptosis, which may be mediated *via* the inhibitory, co-stimulatory molecule B7-H1, soluble factors such as galectin-3, the T cell chemokine attractants VEGF, chemokine (C-C motif) ligand 2 (CCL2), and prostaglandin E2 (202, 207–210), leading to silencing of the immune response and escape immune surveillance in GBM. Contrarily, the accumulation of these pro-tumorigenic TAMs in tumors could trigger the secretion of high levels of pro-inflammatory cytokines, such as RAGE, COX2, and NF-kB (211, 212), contributing to tumor progression and GSC maintenance. Accordingly, targeting innate immune cells and receptors correlated with their secreta, such as macrophage colony stimulation factor receptor (213), have been shown to increase tumor sensitivity to anti-GBM therapy, and could become a promising therapeutic target for GBM.

## Conclusion and Future Directions

Here, we shed light on the knowledge of GSCs that are consistent with the general definition of CSCs, their resistance mechanism, vital signal pathways, the crosstalk between GSCs and their niche, and describe how this can prompt the persistence and progression of these stem cells. We also provide a framework for targeting strategies to GSCs consisting of direct and indirect strategies with some constructive opinions presented towards different targeting strategies in the review. Targeting GSCs provides a tremendous opportunity for revolutionary approaches, and a wide variety of reliable therapeutic strategies have been identified that are being clinically translated to improve the prognosis and therapy of GBM after several decades of stem cell therapy experiments, despite the need to simultaneously meet a variety of challenges, such as how to target GSCs effectively while avoiding impairing normal NSCs and progenitor cells. Accordingly, more authentically useful approaches for the isolation and identification of GSCs, further laboratory and clinical investigations regarding crosstalk between GBM and GSCs are needed to gain insights into maintenance, therapy resistance, and recurrence in tumors, as well as the origin, properties, and progression of GSCs. In the future, efficient design of clinical trials, novel approaches for targeting GSCs, and potent anti-GBM agents that could be used to suppress GSCs and become useful for future clinical applications are urgently needed.

## Author Contributions

XT, RT, and YH conceived the article. XT compiled the review and prepared the draft of the manuscript. PF, RT, YH, ZZ, GL, and YQ reviewed and edited the manuscript. All authors contributed to the article and approved the submitted version.

## Conflict of Interest

The authors declare that the research was conducted in the absence of any commercial or financial relationships that could be construed as a potential conflict of interest.
